# Charge carrier mobility and electronic properties of Al(Op)_3_: impact of excimer formation

**DOI:** 10.3762/bjnano.6.112

**Published:** 2015-05-05

**Authors:** Andrea Magri, Pascal Friederich, Bernhard Schäfer, Valeria Fattori, Xiangnan Sun, Timo Strunk, Velimir Meded, Luis E Hueso, Wolfgang Wenzel, Mario Ruben

**Affiliations:** 1 Institute of Nanotechnology, Karlsruhe Institute of Technology, D-76344 Eggenstein-Leopoldshafen, Germany; 2 Istituto per la Sintesi Organica e Fotoreattivitá, Consiglio Nazionale della Ricerca, I-40129 Bologna, Italy; 3 CIC nanoGUNE Consolider, E-20018 Donostia – San Sebastian, Spain; 4 Nanomatch, Hermann-von-Helmholtz-Platz 1, D-76344 Eggenstein-Leopoldshafen, Germany

**Keywords:** charge carrier mobility, HOMO–LUMO energy levels, photophysical characterization, TFT devices, tris-(1-oxo-1*H*-phenalen-9-olate)aluminum(III)

## Abstract

We have studied the electronic properties and the charge carrier mobility of the organic semiconductor tris(1-oxo-1*H*-phenalen-9-olate)aluminium(III) (Al(Op)_3_) both experimentally and theoretically. We experimentally estimated the HOMO and LUMO energy levels to be −5.93 and −3.26 eV, respectively, which were close to the corresponding calculated values. Al(Op)_3_ was successfully evaporated onto quartz substrates and was clearly identified in the absorption spectra of both the solution and the thin film. A structured steady state fluorescence emission was detected in solution, whereas a broad, red-shifted emission was observed in the thin film. This indicates the formation of excimers in the solid state, which is crucial for the transport properties. The incorporation of Al(Op)_3_ into organic thin film transistors (TFTs) was performed in order to measure the charge carrier mobility. The experimental setup detected no electron mobility, while a hole mobility between 0.6 × 10^−6^ and 2.1 × 10^−6^ cm^2^·V^−1^·s^−1^ was measured. Theoretical simulations, on the other hand, predicted an electron mobility of 9.5 × 10^−6^ cm^2^·V^−1^·s^−1^ and a hole mobility of 1.4 × 10^−4^ cm^2^·V^−1^·s^−1^. The theoretical simulation for the hole mobility predicted an approximately one order of magnitude higher hole mobility than was observed in the experiment, which is considered to be in good agreement. The result for the electron mobility was, on the other hand, unexpected, as both the calculated electron mobility and chemical common sense (based on the capability of extended aromatic structures to efficiently accept and delocalize additional electrons) suggest more robust electron charge transport properties. This discrepancy is explained by the excimer formation, whose inclusion in the multiscale simulation workflow is expected to bring the theoretical simulation and experiment into agreement.

## Introduction

**Figure 1:**
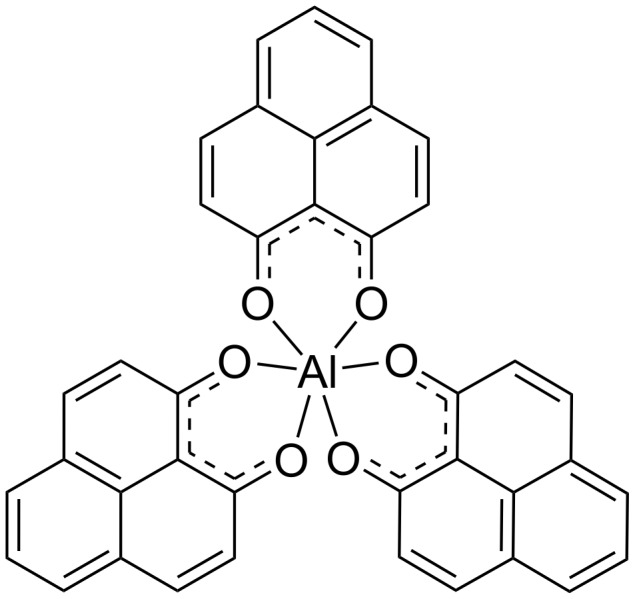
Tris(1-oxo-1*H*-phenalen-9-olate)aluminum(III) (Al(Op)_3_) structure. H atoms are omitted for clarity.

Since the field of organic electronics has emerged, interest in organic semiconductors (OSCs) has substantially increased [1]. The efficiency with which electron and/or holes move within the organic layer is crucial to device performance [2]. Since its first implementation in OLEDs devices [3], the small p-conjugated tris(8-hydroxyquinolinolate)aluminum(III) (Alq_3_) is still the most commonly used and studied electron transport material among the small-molecule-based OSCs [4]. It is mostly chosen because of its integration properties, namely, it can be easily deposited as a thin film and included into devices with a variety of metallic electrodes. In addition, the electron mobility of Alq_3_, which ranges between 10^−5^–10^−6^ cm^2^·V^−1^·s^−1^, is considerably higher than the corresponding hole mobility, measured between 10^−8^–10^−9^ cm^2^·V^−1^·s^−1^ [5–12]. This is a principally important property that makes Alq_3_ a fairly good electron transporting material and, in addition, an intrinsic hole blocking material, which is essential for charge recombination confinement and thereby increasing the efficiency of organic LED devices. As a result of the electron-deficient quinoline ligand, Alq_3_ is characterized by HOMO and LUMO energy levels of ≈−5.95 and ≈−3.0 eV, respectively [13–14]. The LUMO and HOMO energy levels are fundamental parameters for charge transporting materials [15]. In particular, lower LUMO and HOMO energies enable easier reduction of the metal chelate, leading to enhanced electron injection and transport properties, and an increased resistance to oxidization, resulting in an improved hole blocking character. For this very reason we have synthesized and studied the phenalenyl-based alternative, OSC tris(1-oxo-1*H*-phenalen-9-olate)aluminum(III) (Al(Op)_3_) (see Figure 1), which is formed by ligands with an extended aromatic system. The expected result is an increased capability to accept and efficiently delocalize additional electrons, and thus, Al(Op)_3_, should be characterized by both lower HOMO and LUMO energy levels as compared to Alq_3_.

In a recent work, Al(Op)_3_ deposited on a magnetic cobalt substrate was investigated by means of ultraviolet photoemission spectroscopy (UPS) and near-threshold photoemission spectroscopy (NT-PS) [16]. The purpose was to study the spin-dependent properties of the Co/Al(Op)_3_ hybrid interface in comparison with the Co/Alq_3_ hybrid interface [17]. Due to the difference in the aromatic structures of Al(Op)_3_ and Alq_3_, which influences the chemisorption onto the cobalt substrate, it was demonstrated that the Co/Al(Op)_3_ and the Co/Alq_3_ interfaces have different electronic properties. In more detail, two hybrid interface states, which have energies (*E*−*E*_F_) of −0.9 and −1.6 eV were detected in the Co/Al(Op)_3_ interface, whereas in the Co/Alq_3_, a single hybrid interface state at −0.8 was measured. In this work, the potential of the chemical tailoring of the aluminum complexes’ ligands was successfully demonstrated along with the resulting effects on the electronic properties of the hybrid interfaces.

To our knowledge, this is surprisingly the first time that the electronic properties of Al(Op)_3_ are reported. Phenalenyl-based complexes (more specifically lanthanoid phenalenyls) have been previously studied for their peculiar photoluminescence in the gas phase [18–19]. Greisch et al. observed that the alkali metal cationization of Eu(Op)_3_ increases the capability of the ligand 1-oxo-1*H*-phenalen-9-olate to sensitize the europium ion. Furthermore, for lithium and sodium, the enhancement was found to be the most efficient. In these works, 9-hydroxyphenalen-1-one was carefully chosen for its photophysical properties, namely, its high absorption cross section in the condensed phase between near-UV and 475 nm [20] and its phosphorescence at 17,350 cm^−1^, which is characterized by a lifetime of about 25 ms [21]. Furthermore, Van Deun et al. demonstrated that 9-hydroxyphenalen-1-one can form stable complexes with lanthanides and transfer energy to europium in coordination complexes [22].

Many of the aforementioned properties stem either completely or mostly from the chosen ligand’s chemical structure, specifically: the symmetrical geometry, the two oxygen atoms as chelating atoms, and the extended aromatic system. Considering this, we were interested in the possible impact of the given ligand on potential OSC opto-electronic performance.

Intrigued by establishing a direct relation between the chemical structure and the electronic properties of OSCs, we have fully characterized Al(Op)_3_ by means of electrochemical and photophysical techniques in solution to estimate the HOMO/LUMO values as well as in thin films to investigate the solid state properties. Moreover, the aluminum complex has been implemented in organic thin film transistors devices (TFTs) to measure the charge carrier mobility. Finally, an extensive theoretical investigation has been carried out for comparison with the experimental data.

## Results

Al(Op)_3_ was synthesized as previously described [23]. To confirm the purity of the complex, proton and carbon nuclear magnetic resonance (NMR) spectroscopy and mass spectrometry (MS) were carried out and compared with the data reported in previous work [16].

**Figure 2:**
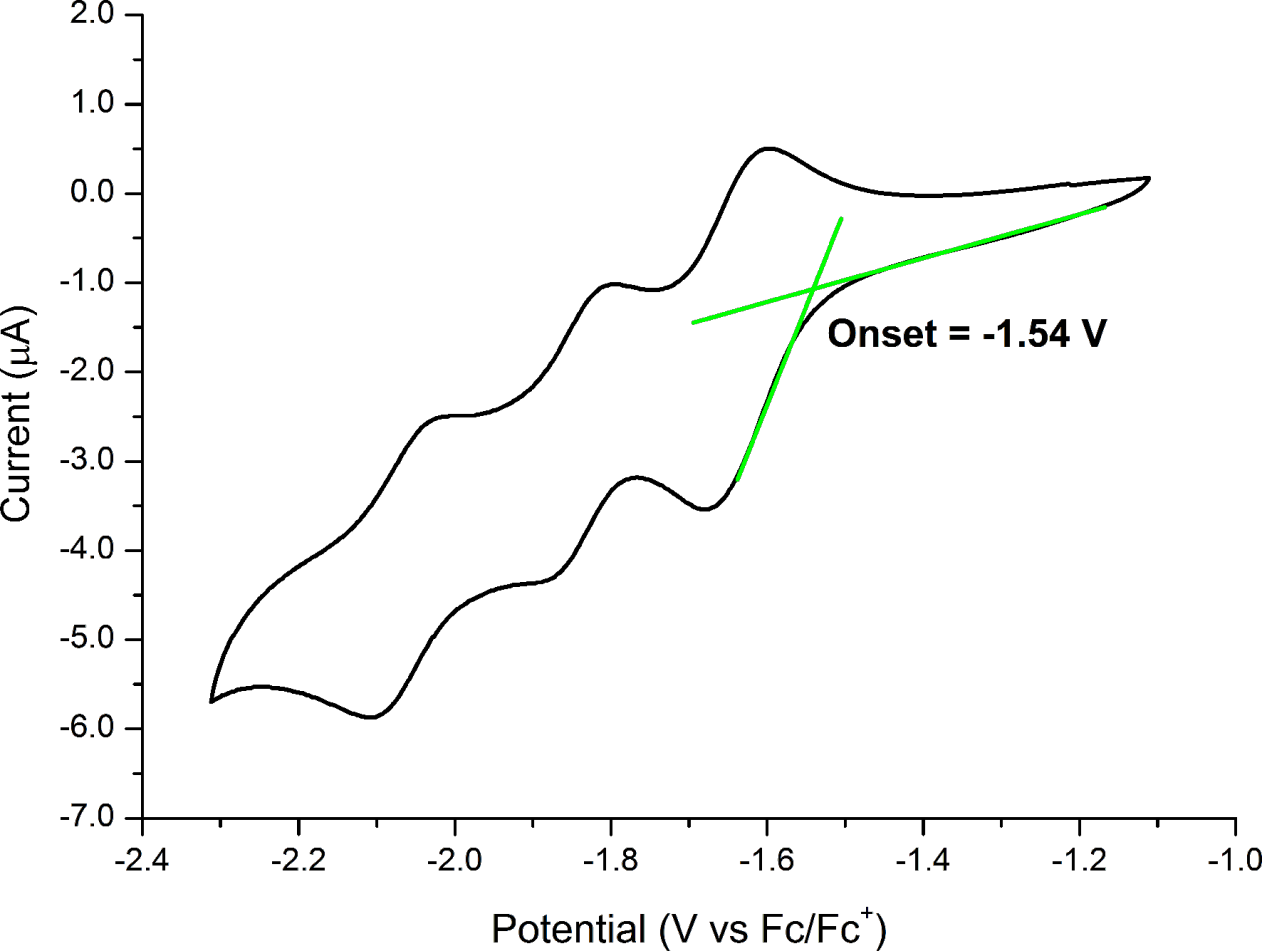
Cyclic voltammogram for Al(Op)_3_ recorded at room temperature in CH_2_Cl_2_ solution using TBAPF_6_ as the electrolyte and ferrocene as an internal standard. Scan rate: 0.1 Vs^−1^. The graphical estimation of the first reduction wave onset is highlighted in green. The LUMO energy level, −3.26 eV, was computed by using the onset of the first reduction wave at −1.54 V [24–25].

Initially, in order to evaluate the potential incorporation of Al(Op)_3_ in organic-based devices, we have estimated its HOMO/LUMO energies by electrochemical and photophysical methods in solution. The electron affinity (EA) was measured by means of cyclic voltammetry and the ionization potential (IP) was determined by the absorption spectrum. In experiment, the IP and the EA are referred to as the HOMO and LUMO energy levels of the molecule, respectively [15,24]. The cathodic cyclic voltammetry of Al(Op)_3_, shown in Figure 2, is characterized by three, quasi-reversible, single-electron transfer processes at −1.63, −1.84 and −2.07 V. The subsequent formation of the mono-, di-, and tri-anion is assumed to occur due to the systematic reduction of each phenalenyl moiety [25]. Since electron transport can be represented as a series of consecutive redox processes, the reversible electrochemical reduction with an adequately high reduction potential is expected to promote the transport of electrons within the organic film [24]. From the onset of the first reduction wave, we have estimated a LUMO energy of Al(Op)_3_ of −3.26 eV [26–27]. Implementing the same procedure, the LUMO energy of Alq_3_ is −3.01 eV. As expected, the extended aromatic system of Al(Op)_3_, which can more efficiently delocalize an additional electron, leads to a lower LUMO energy.

**Figure 3:**
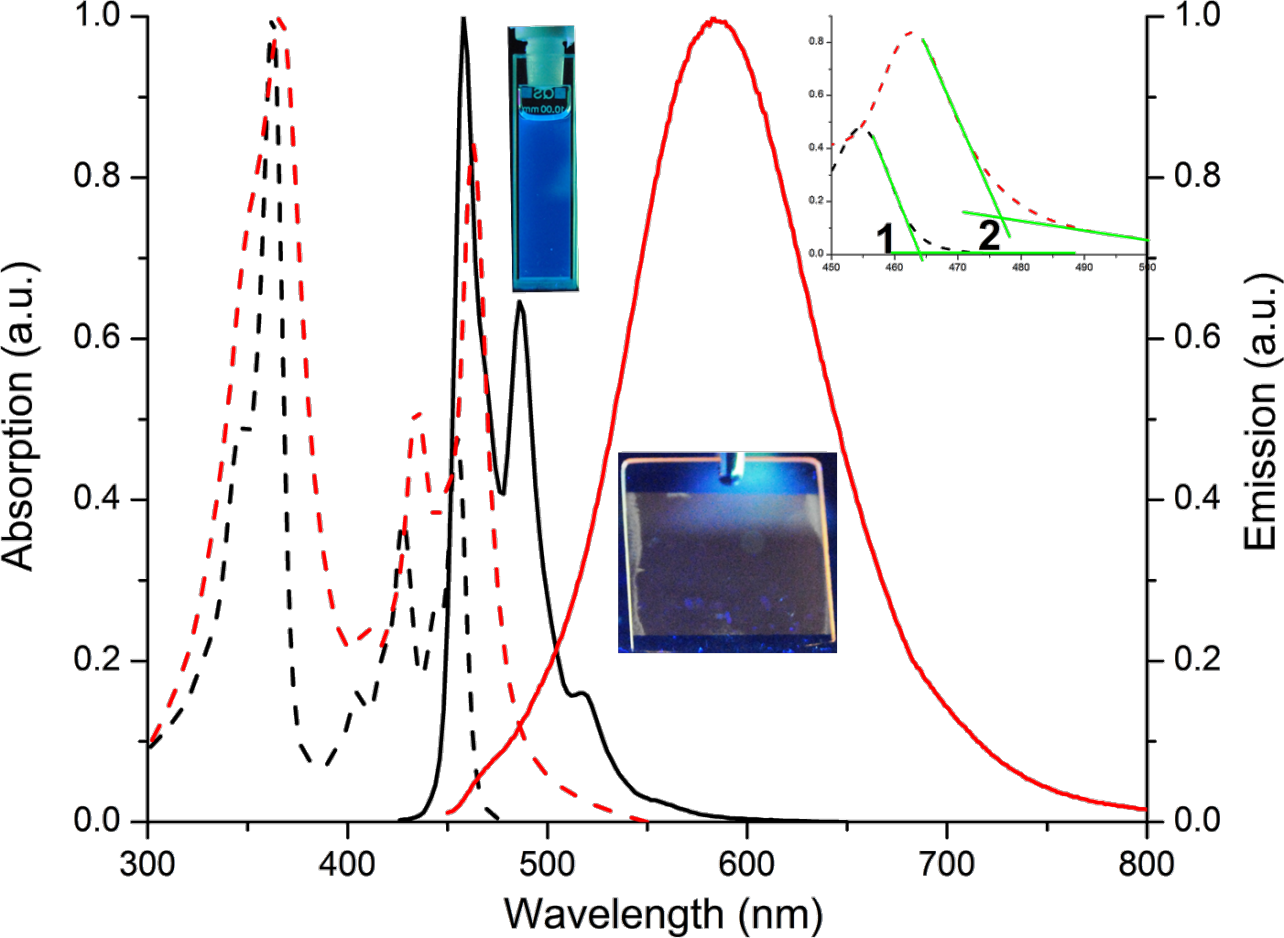
Absorption (dotted line) and emission spectra (solid line) of Al(Op)_3_ in CH_2_Cl_2_ solution (black) and as a thin film on quartz (red). In the absorption spectra, the first band, between 325 and 375 nm (ε_362_ = 90.7 × 10^3^ M^−1^cm^−1^), is associated with π→π* transitions. The second structured band, between 400 and 475 nm (ε_454_ = 43.6 × 10^3^ M^−1^cm^−1^), is attributed to n→π* transitions. In the inset the region of the first absorption band and the graphical estimation of the onset of the bands are illustrated. The onset of Al(Op)_3_ in solution is 464 nm (1) and in the thin film is 476 nm (2). The respective optical HOMO–LUMO band gaps converted to eV are 2.67 eV and 2.60 eV. The emission spectra were recorded with an excitation wavelength of 350 nm. The excimer formation is clearly noticeable from the difference in the emission peaks in solution and in the thin film. Photographs of the samples under UV irradiation are included to show the visible color change.

Using the onset of the first band in the absorption spectrum in solution, as shown in Figure 3, an optical HOMO–LUMO gap of 2.67 eV was determined. Thus, the HOMO energy of Al(Op)_3_ was calculated to be −5.93 eV, in fairly good agreement with the HOMO energy previously estimated by UPS on 1.5 and 5 nm films of Al(Op)_3_ deposited on cobalt which are −6.5 and −6.9 eV, respectively [16]. According to the same procedure, the HOMO–LUMO gap and the HOMO energy of Alq_3_ were estimated to be 2.82 and −5.83 eV, respectively. As a result of the lower LUMO energy of Al(Op)_3_ compared to Alq_3_, the injection of electrons should be not only possible, but enhanced, as a consequence of the reduced mismatch with a cathode such as aluminum with a work function of Φ ≈ 4.3 eV. In addition, considering the similar trend in the HOMO energies of Al(Op)_3_ and Alq_3_, we could, based on this single molecule energy analogy, assume that Al(Op)_3_, when implemented in a device, would prevent hole diffusion in the same manner as Alq_3_.

In order to confirm the thermal stability of Al(Op)_3_, the complex was deposited by thermal evaporation onto a quartz substrate forming an 80 nm thin film and the photophysical properties were measured. The almost identical profiles of the absorption spectra in CH_2_Cl_2_ solution and in the thin film (see Figure 3) confirm that the complex was successfully grown onto the quartz substrate. Due to the solvatochromic effect, the absorption bands have different relative intensities and result in a slight shift [28–29]. Consequently, the HOMO–LUMO gap in the thin film is 2.60 eV, which is slightly narrower than in solution.

**Figure 4:**
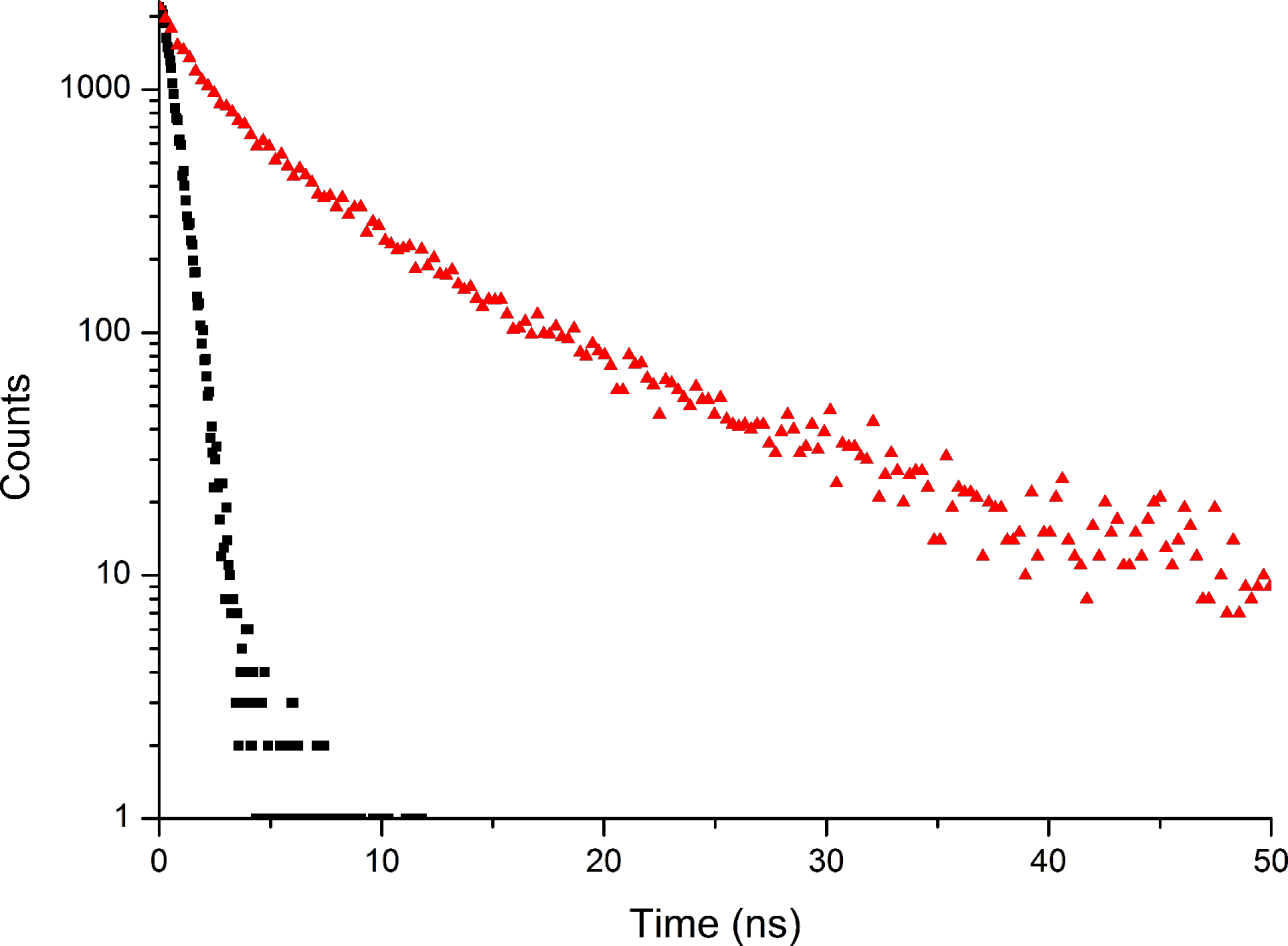
Luminescence decay in CH_2_Cl_2_ solution (black) and as a thin film on quartz (red). In solution, a mono-exponential decay is observed with a lifetime τ ≈ 0.7 ns, while in the thin film, a multi-exponential decay is observed with an average lifetime τ ≈ 7.1 ns.

We have further investigated Al(Op)_3_ by measuring the steady state emission spectra in solution and as a thin film at room temperature (see Figure 3). The emission in a CH_2_Cl_2_ diluted solution is characterized by a structured band with a maximum at 458 nm. Conversely, the emission in the thin film is dominated by a broad band peaked at 583 nm. To explain this, we have presumed excimer formation within the thin film. Normally, an excimer is caused by a charge-transfer interaction between an electronically excited species and a ground state molecule [30–31]. Often, the excimer possesses observable properties quite distinct from those of the single molecule [30–31]. The photophysical properties in solution and in thin film are summarized in Table 1 and are consistent with excimer formation. In more detail, Al(Op)_3_ in solution is characterized by a photoluminescence quantum yield (Φ) of 0.027 and a lifetime (τ) of 0.7 ns. In the form of a thin film, the quantum yield (Φ) is considerably lower, 0.014, and the lifetime (τ) is an order of magnitude longer, 7.1 ns. The lifetimes were calculated by the luminescence decays, in solution and in the thin film, as shown in Figure 4.

**Table 1:** Photophysical properties of Al(Op)_3_ in CH_2_Cl_2_ solution and in the thin film at room temperature.

Al(Op)_3_	λ_abs,max_ [nm]	λ_emi,max_ [nm]	Φ	τ [ns]

Solution	362	458	0.027	0.7
Thin film	366	583	0.014	7.1

### Field effect mobility in TFT devices

**Figure 5:**
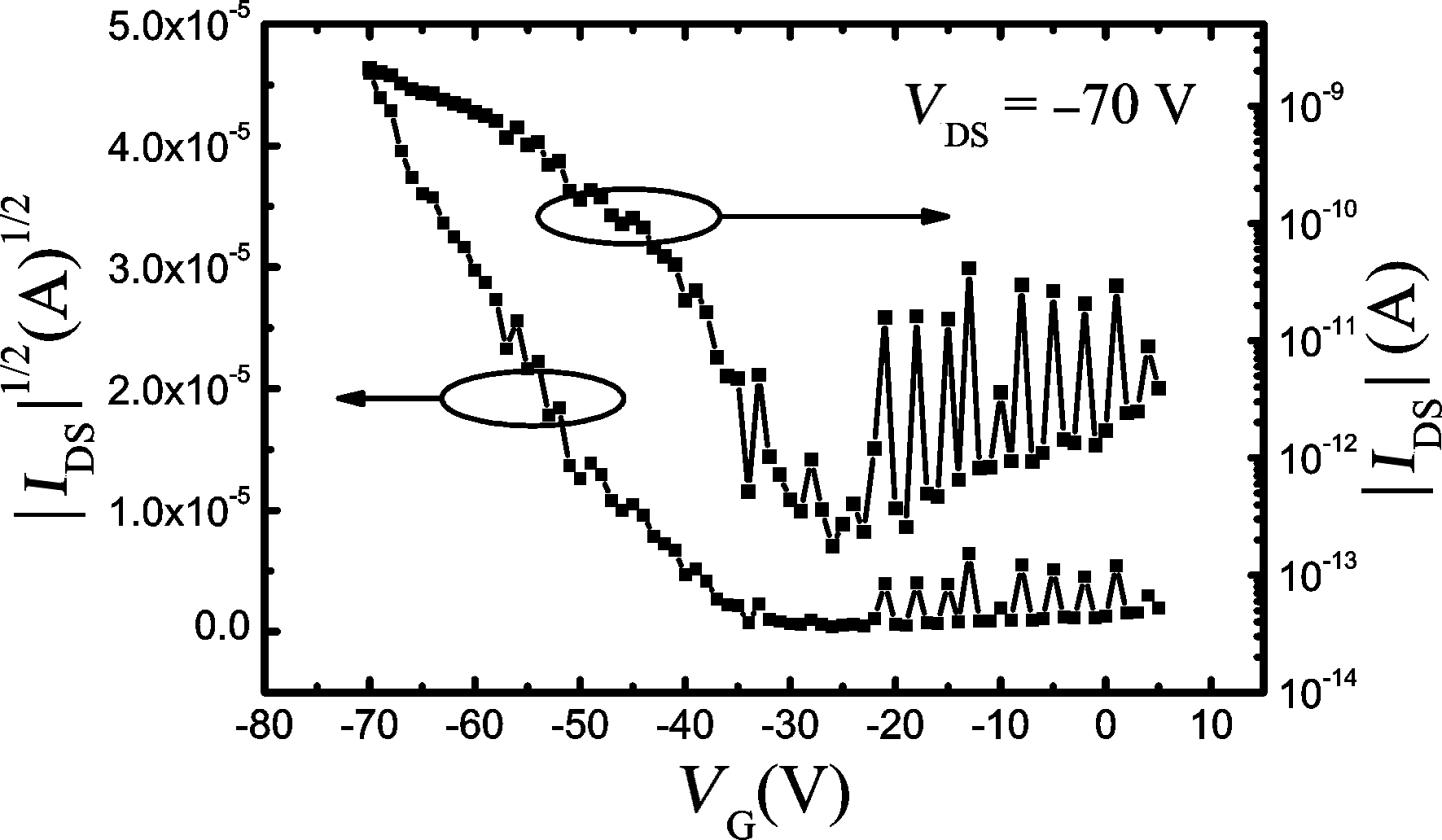
Transfer curve of the Al(Op)_3_-based TFT with a channel length of 100 μm. In this figure, *I*_DS_ and *V*_DS_ are the source–drain current and voltage and *V*_G_ refers to the gate voltage. The hole mobility, extrapolated by the transfer characteristics, ranges between 0.6 × 10^−6^ and 2.1 × 10^−6^ cm^2^·V^−1^·s^−1^.

From the electrical characteristics measured in a field-effect transistor (FET) configuration, it is possible to obtain the charge carrier mobility of electrons and holes [32]. Therefore, in order to measure the field-effect mobility of Al(Op)_3_, thin film transistors (TFTs) based on Al(Op)_3_ were fabricated. A series of Al(Op)_3_-based TFTs were built with channel lengths ranging from 10 to 100 μm and with channel width/length (W/L) ratios of 20000/10, 20000/20, 10000/50, and 5000/100. From the electrical characterization of the TFT devices, the transfer curves, which yield the charge carrier mobility, were determined. As an example, in Figure 5, the transfer curve relative to the TFT device with a channel length of 100 μm is shown. The curve clearly outlines a p-type transistor behavior of the device [33–35], and the on/off current ratio calculated from this curve is greater than 10^4^. As a result of the electrical characterization of four Al(Op)_3_-based TFT devices, we have estimated the field effect mobility from the slope of the high-voltage section of the transfer curve by the equation for the saturation regime [34–35]. The hole mobility was found to range between 0.6 ×10^−6^ and 2.1 × 10^−6^ cm^2^·V^−1^·s^−1^, and the threshold voltage between −35 and −45 V.

**Figure 6:**
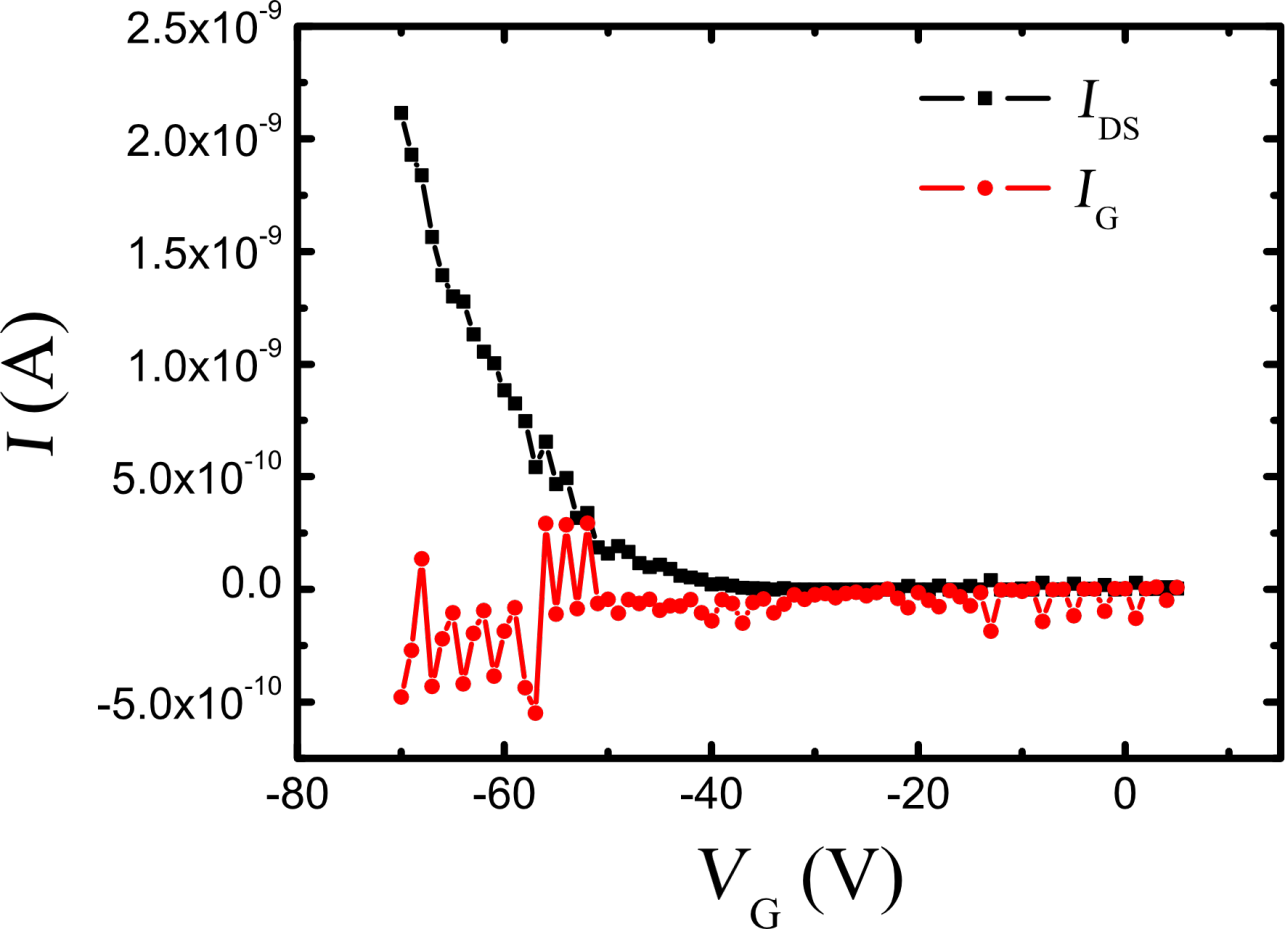
Comparison of the source–drain current (*I*_DS_) and the leakage gate current (*I*_G_) from the transfer characterization of the Al(Op)_3_-based TFT with a channel length of 100 μm. The source–drain bias, *V*_DS_, for this measurement is −70 V. *I*_SD_ currents are far higher than *I*_G_ in the high voltage regime, indicating that the mobility calculated from the transfer curves is reliable and not impacted by the leakage current.

In the transfer characteristics (see Figure 6), the source–drain currents (*I*_SD_) are far higher than the leakage current (gate current, *I*_G_) in the high voltage regime in which the mobility has been calculated [34–35]. Therefore, we can conclude that the measured hole mobility from the transfer curves is reliable and not substantially impacted by the leakage current [34–35].

In all the measurements performed on the Al(Op)_3_-based TFTs no obvious electron mobility was detected. This was indeed expected, due to the excimer formation observed in the solid state photo-physical characterization. Excimers in the organic thin film or at the organic/organic interface (exciplexes) act as electron traps, and as a result, the electron mobility can be lowered until the point of suppression (i.e., below the sensitivity of the instrumentation).

### Theory of HOMO–LUMO level charge mobility

In order to shed more light on the problem at hand, we performed density functional-based [36–38] calculations for both a single molecule in vacuum and molecules embedded in an explicit matrix [39] and compared them to a de facto standard in the field, namely, Alq_3_.

**Figure 7:**
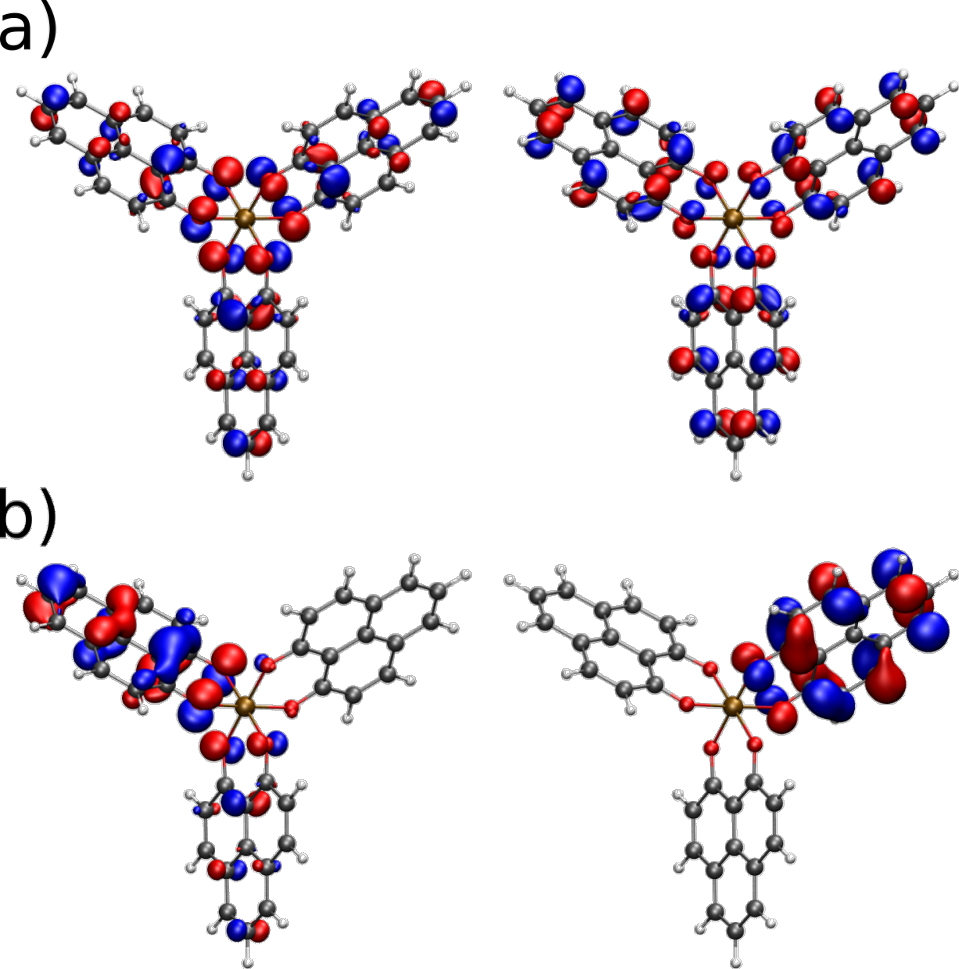
a) HOMO (left) and LUMO (right) orbitals of Al(Op)_3_ calculated with TURBOMOLE [36] on a B3-LYP [37]/SV(P) [38] level of theory in vacuum. b) The same orbitals calculated in the explicit matrix, represented by a self-consistently evaluated cloud of point charges. The electrostatic interaction with the environment leads to the localization of the frontier orbitals.

**Figure 8:**
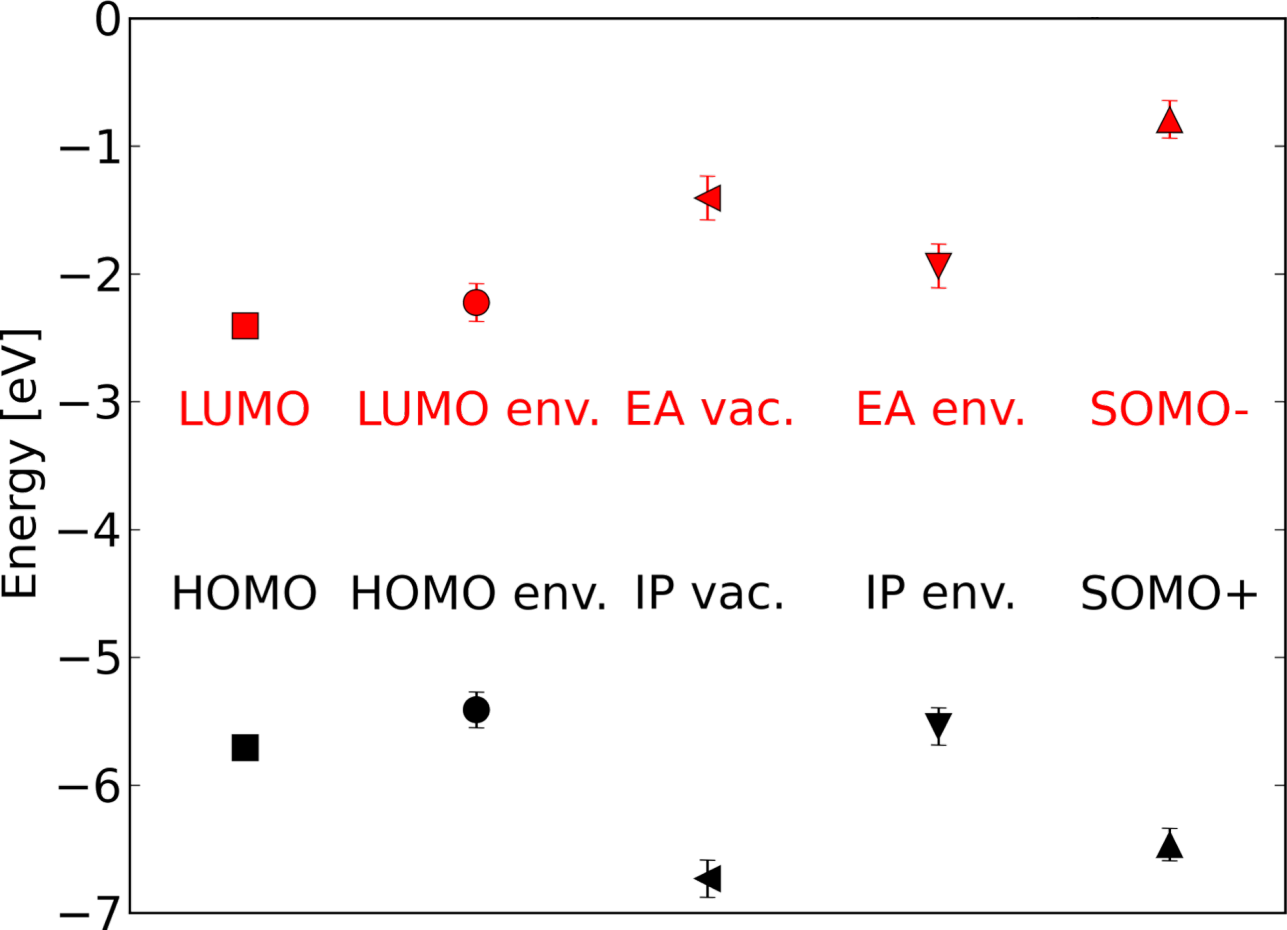
Energy levels of Al(Op)_3_ calculated with different conditions, namely, HOMO and LUMO in vacuum (vac.) and in a self-consistent electrostatic environment (env.), with electron affinity (EA) and ionization potential (IP) in vacuum and environment and SOMO + and − orbitals in vacuum.

Structurally, Al(Op)_3_ is formed by symmetric ligands that bind to the Al^3+^ ion via oxygen donor atoms only. As a consequence, the electron density of the HOMO/LUMO levels (shown in Figure 7a) is equally distributed over the three chelating moieties. In contrast, in Alq_3_ oxygen and nitrogen atoms connect to the metal ion in symmetrically non-equivalent positions. Hence, the HOMO/LUMO energies of Alq**_3_** are localized mainly on one ligand [40]. The results for HOMO/LUMO levels of Al(Op)_3_ calculated with DFT (but on different level of theory) are shown in Figure 8. We calculated the ionic and electronic ground state of the molecule in vacuum and extracted the HOMO and the LUMO energies of −5.71 eV and −2.41 eV with a gap of 3.3 eV (376 nm). Compared to that, the HOMO and LUMO energies of the widely studied Alq_3_ molecule are −5.14 eV and −1.91 eV with a gap of 3.22 eV (385 nm).

After performing the single molecule vacuum calculations, we set up calculations for molecules in a matrix, which gives the molecular properties in terms of distributions, with both center and width, rather than unique numbers. The calculation of HOMO and LUMO levels in the condensed phase from an atomistic morphology using the quantum patch method without additional charges [39] leads to −5.41 eV for the HOMO energy and −2.22 eV for the LUMO energy. The calculation of the ionization potential and the electron affinity by the self-consistent evaluation of the total energies of charged and uncharged molecules in the condensed phase leads to IP = 6.73 eV and EA = 1.41 eV in vacuum and IP = 5.54 eV and EA = 1.94 eV in environment. Additional charges in the system, which can be considered as SOMO+ and SOMO− orbital energies, lead to values of −6.47 eV for the oxidized state (hole) and −0.79 eV for the reduced state (electron). The self-consistently evaluated HOMO and LUMO levels in an Alq_3_ matrix are −5.07 eV and −1.80 eV, respectively. The vacuum IP is 6.54 eV and the vacuum EA is 0.64 eV. In the matrix, the IP is 5.34 eV and the EA is 1.5 eV. The SOMO+ and SOMO− orbitals are at −6.47 eV and 0.19 eV, respectively. All calculations were performed with the B3LYP [37] exchange correlation functional and an SV(P) [38] basis set.

The comparison between the calculated dipole moments of Al(Op)_3_ and Alq_3_ show a much weaker dipole moment of 0.10 D for Al(Op)_3_ as compared to the vacuum dipole moment of 4.46 D for Alq_3_. On the other hand, in the matrix, the dipole moment for Al(Op)_3_ is 1.51 ± 0.60 D and 5.55 ± 0.91 D for Alq_3_. The increase of the dipole moment of Al(Op)_3_ is much more dramatic, indicating a break in the intrinsic, vacuum symmetry, which is reflected in the orbital localization and is clearly observable in Figure 7b. As we treated the molecules in the matrix to be rigid, the deviations between the vacuum and matrix dipole moments arise from induction and polarization effects present only in the matrix, which influence the energy disorder.

Furthermore, we calculated the width of the local density of states for additional charges (if a Gaussian shape is assumed, this is referred to as energy disorder, σ), the mean electronic coupling between molecules, <*J*^2^*r*^2^>, the mean number of neighbors, *M*, and the reorganization energy, λ. These results are shown in Table 2. These microscopic parameters can be used to calculate the charge carrier mobility [41]:

[1]
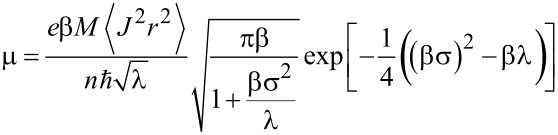


where *e* is the electric charge and 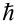 is the Plank constant. The reciprocal temperature, β, is defined as 
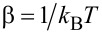
, where *k*_B_ is the Boltzmann constant and *T* is the temperature, held constant at 290 K for these calculations. This analytic expression yields a zero-field charge carrier mobility of 1.4 × 10^−4^ cm^2^·V^−1^·s^−1^ for holes and 9.5 × 10^−6^ cm^2^·V^−1^·s^−1^ for electrons, which can be compared to Alq_3_ having a hole mobility of 3.3 × 10^−8^ cm^2^·V^−1^·s^−1^ and an electron mobility of 9.2 × 10^−8^ cm^2^·V^−1^·s^−1^. The differences between AlOp_3_ and Alq_3_ mainly arise from different dipole moments, which lead to a higher energy disorder, σ, for Alq_3_. Al(Op)_3_ reacts less strongly on charging, leading to a smaller reorganization energy, λ, than for Alq_3_. The slightly higher electronic coupling, *<J^2^r^2^>*, of Alq_3_ is compensated by a smaller number of neighbors, *M*. The calculated HOMO and especially LUMO levels are comparable to those of Alq_3_ (−5.14 and −1.91 eV without environment), making electron injection in general plausible. The simulated hole mobility is about one order of magnitude higher than the mobility of 9.5 × 10^−6^ cm^2^·V^−1^·s^−1^ measured in experiment. This overestimation is considered reasonable as our morphologies consist of rigid Al(Op)_3_ molecules, which leads to a slight underestimation of the energy disorder, and therefore, a calculated mobility that is too high. Further, it is known from previous studies [39] that the experimental results for charge mobility can vary by up to two orders of magnitude, depending on the details of the experimental setup.

**Table 2:** Microscopic parameters and charge carrier mobility for Al(Op)_3_ and Alq_3_ used as input in Equation 1.

	σ [eV]	<*J*^2^*r*^2^> [eV^2^Å^2^]	*M*	λ [eV]	µ [cm^2^·V^−1^·s^−1^]

Al(Op)_3_ hole transport	0.142	4.42 × 10^−3^	7.9	0.055	1.4 × 10^−4^
Al(Op)_3_ electron transport	0.167	7.04 × 10^−3^	7.9	0.060	9.5 × 10^−6^
Alq_3_ hole transport	0.187	1.10 × 10^−2^	5.6	0.336	3.3 × 10^−8^
Alq_3_ electron transport	0.184	1.34 × 10^−2^	5.6	0.285	9.2 × 10^−8^

Nonetheless, the lack of electron mobility in the experiment can be explained by the observation of excimer formation in Al(Op)_3_, as explained above. Accounting for the excimer formation in the charge mobility workflow is expected to considerably improve the agreement between theory and experiment.

## Discussion

The initial estimate, which due to the extended aromatic system of the ligand Al(Op)_3_ should be characterized by lower HOMO/LUMO energy levels as compared to Alq_3_, has been proven by experimental and theoretical methods. The HOMO/LUMO energy levels of Al(Op)_3_ are: −5.93 and −3.26 eV experimental, −5.71 and −2.42 eV theoretical in vacuum and −5.41 and −2.22 eV theoretical in the assumed amorphous film. The HOMO/LUMO energy levels of Alq_3_ are: −5.83 and −3.01 eV experimental, −5.14 and −1.91 eV theoretical in vacuum and −5.07 and −1.80 eV theoretical in the assumed amorphous film. As a consequence, the electron injection from a cathode should be more efficient in Al(Op)_3_ layers compared to Alq_3_. Nonetheless, the Al(Op)_3_ HOMO energy is high enough to prevent hole diffusion. The major drawback of the extended and flat aromatic system, which can accept and efficiently delocalize up to three electrons at relatively low reduction potentials (as shown by the cyclic voltammetry experiment in Figure 2), is the formation of excimers in the solid state (see Figure 3). Excimers or exciplexes are known to prevent electron diffusion, acting as traps in the organic layer. This is confirmed by the charge carrier mobility measured in TFT devices where a hole mobility between 0.6 × 10^−6^ and 2.1 × 10^−6^ cm^2^·V^−1^·s^−1^ was measured. However, no electron mobility was detected. Nevertheless, the experimentally measured Al(Op)_3_ hole mobility is two orders of magnitude higher than the reported hole mobility of Alq_3_, which ranges between 10^−8^ and 10^−9^ cm^2^·V^−1^·s^−1^. This trend is confirmed by simulations produced by the newly developed, multiscale, charge mobility workflow. Here, the calculated hole mobility for Al(Op)_3_ and Alq_3_ is 9.5 × 10^−6^ and 9.2 × 10^−8^ cm^2^·V^−1^·s^−1^, respectively. The initial assumption of the easier delocalization and transport of electrons (sustained by the extended aromatic system of the ligand forming the coordination complex Al(Op)_3_) is confirmed by the electron mobility calculation. The predicted electron mobility for Al(Op)_3_ and Alq_3_ is 1.4 × 10^−4^ and 3.3 × 10^−8^ cm^2^·V^−1^·s^−1^, respectively. While the calculations exclude increased thin film disorder as a contributing factor to the experimentally observed low electron mobility, in order to obtain accurate and realistic predictions on the charge mobility, taking the formation of excimers in the thin film into account is of a primary importance.

## Conclusion

We have synthesized and characterized a novel, Al-based, metallo-organic molecule as an alternative to the de facto, industry standard, electron transporting material, Alq_3_. The initial assumptions based on the extended aromatic structure of Al(Op)_3_ and confirmed by the theoretical DFT-based simulations (1.4 × 10^−4^ cm^2^·V^−1^·s^−1^ electron mobility and 9.5 × 10^−6^ cm^2^·V^−1^·s^−1^ hole mobility) indicated that the material would be a good Alq_3_ alternative. However, excimer formation, as observed in the photoluminescence experiments in the solid state, may play the most decisive role in disrupting the electron flow through the deposited thin films. Indeed, this is confirmed by Al(Op)_3_-based TFTs devices, in which a hole mobility between 0.6 × 10^−6^ and 2.1 × 10^−6^ cm^2^·V^−1^·s^−1^ was measured, whereas the electron mobility could not be determined. As such, including the excimeric effect into future multiscale simulations seems to be of great importance. The fact that the hole mobility in both theory and experiment as compared to Alq_3_ is considerably higher by two to four orders of magnitude implies that this material cannot be considered as a good hole blocking layer material. As such, it could be used in organic electronic devices only together with an additional explicit hole blocking material layer.

## Experimental

Al(Op)_3_ was synthesized according to the procedure reported in [16]. The analytical characterization data of Al(Op)_3_ can also be found in [16].

### Characterization in solution

Cyclic voltammetry was performed using an Autolab PGSTAT10 potentiostat in a three-electrode single-compartment cell with a glassy carbon working electrode, an Ag/AgCl pseudo-reference electrode and a platinum wire as an auxiliary electrode, in an inert argon atmosphere. Tetrabutylammonium hexafluorophosphate (TBAPF_6_) was used as a supporting electrolyte (0.1 M) and CH_2_Cl_2_ was used as the solvent. The concentration of the samples was 1.0 × 10^−4^ M, and the solutions were degassed with argon prior to the measurements. A scan rate of 100 mV·s^−1^ was employed. Ferrocene was used as an internal standard to calculate the corrected redox potential. Absorption and emission spectra were acquired at room temperature for diluted CH_2_Cl_2_ solutions (8.0 × 10^−6^ M) on a Cary 500 Scan UV–vis–NIR spectrophotometer and a Cary Eclipse fluorescence spectrophotometer using a 1 cm quartz cell. The photoluminescence quantum yield was computed using rhodamine 6G as reference [42–43].

### Characterization in thin film

The evaporation of the samples on quartz substrates was carried out using an Edwards Auto 306 evaporator equipped with a high vacuum chamber (10^−6^ mbar) and a frequency thickness monitor (FTM) to check the evaporation rate. The deposition rate was 0.5 nm∙s^−1^ with a final thickness of 80 nm. The solid state absorption spectra were recorded on a Perkin-Elmer Lamba 900 UV–vis–NIR spectrophotometer, the photoluminescence spectra of Alq_3_ and Al(Op)_3_ were acquired on a Spex Fluorolog 2. The photoluminescence quantum yield in solid state was estimated by the absolute method using an integrating sphere [44]. The lifetimes were obtained on a time-correlated single photon counter (TCSPC) equipped with a NanoLED source and a Horiba Jobin-Yvon Fluorohub for the data elaboration.

### TFT fabrication and characterization

The Al(Op)_3_-based TFTs were fabricated with a bottom-gated bottom contact geometry. In these devices, highly doped p-type Si, which has a 150 nm thermally grown SiO_2_ layer on the top, was employed as the substrate and as the bottom gate electrode. The source and drain electrodes were patterned on the Si–SiO_2_ substrates by electron beam lithography (Raith 150). These electrodes were deposited under high vacuum (Oerlikon evaporator) with an architecture composed of a 1.2 nm Ti bottom part and a 42 nm Au top part. Before depositing the organic layer, the substrate was cleaned by oxygen plasma for 5 min and modified with trichloro(octadecyl)silane (OTS, ≥90%, Aldrich) by the vapor-phase modification method. This process was carried out in a vacuum oven placed inside a glove box with an inert N_2_ atmosphere (H_2_O and O_2_ concentration <0.1 ppm). Finally, a 40 nm Al(Op)_3_ layer was deposited on the OTS-modified substrate in a ultra-high vacuum evaporator (a dual chamber, Theva system). The Al(Op)_3_-based TFTs were characterized in a Lake Shore probe station with a Keithley 4200 semiconductor characterization system. All the TFT electrical measurements were carried out under vacuum at room temperature and in the dark to avoid decomposition of the organic material.

### Theoretical Method

The charge carrier mobility was calculated by means of the newly developed, multiscale, charge mobility workflow [39,41,45] for both Al(Op)_3_ and Alq_3_ in order to gain a detailed understanding of the differences in the electronic structure and microscopic properties between these two materials.

The simulation of the charge mobility requires coupling of macroscopic system properties, such as the intrinsic bulk mobility, temperature, applied bias voltage, etc., with the microscopic (often local) properties, such as energy disorder, intermolecular electronic coupling, reorganization energy, etc*.* Thus, this work thereby constitutes one of the quintessential multiscale problems. These properties by themselves require corresponding, often sophisticated and mutually very different, description formalisms. This is indicated by the term “multiscale” itself, as this formalism describes phenomena existing on vastly different time and length scales.

Atomistic morphologies were generated with a Monte Carlo-based, simulated annealing method [46]. The microscopic properties, such as energetic disorder and electronic coupling between the molecules and reorganization energies, were calculated with the quantum patch method as described in [39]. These microscopic parameters were used in an analytic, Marcus-rate-based [45], effective medium approach [38], in order to estimate the charge carrier mobility for electrons and holes in these materials for zero-field and low-carrier concentrations. All quantum chemical calculations were adapted directly into the multiscale workflow, as well as any additional quantum chemical characterization were performed with TURBOMOLE [36] on a B3-LYP [37]/SV(P) [38] level of theory. For the calculation of reorganization energy, a def2-TZVP [47] basis set was used.
